# Relationship between observation time and detection rate of focal lesions in Esophagogastroduodenoscopy: a single-center, retrospective study

**DOI:** 10.1186/s12876-024-03157-3

**Published:** 2024-02-15

**Authors:** Li Dong, Xiaodan Zhang, Yuting Xuan, Peiling Xiong, Yumei Ning, Bing Zhang, Fan Wang, Qiu Zhao, Jun Fang

**Affiliations:** 1 Department of Gastroenterology, Zhongnan Hospital of Wuhan University, 169 Donghu-ro, Wuchang-gu, 430071 Wuhan, China; 2 Department of Cardiology, Zhongnan Hospital of Wuhan University, Wuhan, China

**Keywords:** Esophagogastroduodenoscopy, Quality indicators, Time factors

## Abstract

**Background:**

Current studies have shown that longer observation time can improve neoplastic detection rate. This study aimed to clarify whether endoscopists with longer observation times can detect more focal lesions.

**Methods:**

Based on the mean examination time for Esophagogastroduodenoscopy (EGD) without biopsy, endoscopists were divided into fast and slow groups, and the detection rate of focal lesions was compared between the two groups. Univariate analysis, multivariate analysis and restricted cubic spline were used to explore the factors of focal lesion detection rate.

**Results:**

Mean examination time of EGD without biopsy was 4.5 min. The cut-off times used were 5 min. 17 endoscopists were classified into the fast (4.7 ± 3.6 min), and 16 into the slow (7.11 ± 4.6 min) groups. Compared with fast endoscopists, slow endoscopists had a higher detection rate of focal lesions (47.2% vs. 51.4%, *P* < 0.001), especially in the detection of gastric lesions (29.7% vs. 35.9%, *P* < 0.001). In univariate and multivariate analyses, observation time, patient age and gender, expert, biopsy rate, and number of images were factors in FDR. There is a nonlinear relationship between observation time and FDR.

**Conclusion:**

Longer examination time improves the detection rate of focal lesions. Observation time is an important quality indicator of the EGD examination.

**Supplementary Information:**

The online version contains supplementary material available at 10.1186/s12876-024-03157-3.

## Introduction

Gastric cancer is the fifth most common malignancy and the fourth leading cause of cancer death in the world [1]. Esophagogastroduodenoscopy (EGD) is a common screening and diagnostic modality for gastrointestinal malignancy [2]. EGD has a high negative predictive value, and its false-negative rate is estimated to be between 10% and 20% [3–7]. Missed cancers are detected during follow-up endoscopy. Missed diagnosis of cancer during endoscopy is associated with several factors modifies, including missed lesions, inadequate follow-up lesions, inadequate monitoring of precancerous lesions, and the development of new tumors [8]. The risk of missed cancer is associated with the quality of EGD. High-quality endoscopy can improve the detection rate of early-stage gastric cancer and reduce cancer-related mortality. Endoscopic quality indicators include premedication, photo documentation, reporting, observation time, biopsy rate/protocol, endoscopy education, image-enhanced endoscopy, artificial intelligence [9]. Multiple observation studies have demonstrated that observation time to be an important and independent indicator of endoscopic quality [3, 10, 11]. Prolonged inspection time (> 1 min per centimeter) in Barrett’s esophagus increases the detection rate of high-grade dysplasia or adenocarcinoma [12]. Using 7 min as a cut-off time, slow endoscopists were more likely to detect high-risk lesions and neoplastic lesions than fast endoscopists [3]. The examination time was defined from the time the endoscope reached into the duodenum to the time it was withdrawn, using 3 min as the cut-off time, a higher proportion of neoplasms were detected by slow endoscopists compared to fast endoscopists [11]. Current studies have shown that longer observation time can improve neoplastic detection rate, but there are no studies on the relationship between observation time and overall focal lesion detection rate (FDR), and the effect of observation time on the detection rate of lesions in different parts of the upper gastrointestinal tract. Therefore, we investigated the observation time and lesion detection rate of each endoscopist. This study aimed to clarify whether endoscopists with longer observation times can detect more focal lesions, and shows the effect of observation time on the detection rate of different parts of the upper gastrointestinal tract. Assess the relationship between observation time and focal lesion detection.

## Methods

### Study design

#### Participants

In this single-center retrospective observational study, we reviewed a database of consecutive examinees who underwent outpatient EGD at the Endoscopy Center of Zhongnan Hospital of Wuhan University from January 2020 to June 2023. The exclusion criteria were as follows: patients < 18 years old, patients with a history of gastrointestinal surgery, therapeutic endoscopy, surveillance endoscopy, patients receiving oral anticoagulants regularly, patients with serious organic diseases of the heart, lung, liver or kidney, gastric retention or excess fluid in the stomach, performed by trainee, incomplete data. Informed consent was obtained from each patient included in the study. The study protocol was approved by the Ethics Committee of Zhongnan Hospital of Wuhan University.

#### Endoscopists and EGD examination

The endoscopists participating in this study had independently completed over 1,000 EGD examinations. An endoscopist with more than 10 years of experience in endoscopy is defined as expert [13]. Consistent with previous research methods [3, 11], Endoscopists were divided into fast and slow groups based on the mean observation time for EGDs without a biopsy. Observation time is defined as the time from capturing the first image in the pharynx to removing the endoscope. It is automatically measured by the endoscopic system and recorded in a database. All EGDs were performed using GIF-H260 and GIF-H290 endoscopies (Olympus Optical Co. Ltd., Tokyo, Japan). When using endoscopes, conventional white-light imaging and narrow-band imaging are used alternately. Narrow-band imaging was used during withdrawal from the esophagus. If suspicious lesions are found, narrow-band imaging should be used for further observation, and a biopsy should be performed as indicated. During EGD, we usually capture 40–60 images and the digital images are automatically saved in the endoscopic system. Before endoscopy, pharyngeal anesthesia is performed with a solution of lidocaine. Intravenous anesthesia is given at the patient’s request. According to the guidelines regarding sedation for gastroenterological endoscopy, midazolam is given intravenously and pethidine hydrochloride is given in patients with insufficient sedation.

#### Definitions and outcomes

The primary outcome was the overall focal lesion detection rate. The FDR was calculated as the proportion of EGD in which at least one of the focal lesions were detected. The focal lesions including Barrett’s esophagus, benign tumor of the esophagus, esophageal cancer, reflux esophagitis, esophageal candidiasis, esophageal ectopic gastric mucosa, hiatal hernia, esophageal diverticulum, gastric ulcer, gastric polyps, advanced stages of atrophic gastritis [14], intestinal metaplasia, submucosal tumors of the gastric, gastric cancer, extra gastric compression, gastric diverticulum, duodenal ulcer, duodenal polyps, duodenal diverticulum and submucosal tumors of the duodenal. Secondary outcomes included detection rate of esophageal, gastric and duodenal focal lesions, endoscopic biopsy rate.

#### Statistical methods

The quantitative data are presented as mean ± standard deviation and compared using Student’s t-test if normally distributed, and otherwise described as median (interquartile range, IQR) and compared using the Mann-Whitney U test. We performed the Kolmogorov-Smirnov test for normality. Categorical variables were compared using the chi-squared test. We used the Spearman correlation coefficient to measure the relationship between examination time and lesion detection rate by endoscopists. Simple and multivariate logistic regression analysis was used to identify independent predictors for detecting focal lesions. The correlation between observation time and FDR was evaluated on a continuous scale using a restricted cubic spline based on a multivariate logistic regression model. All statistical analyses were performed using R software (version 4.2.2) and SPSS 26.0 (SPSS Inc., Chicago, III, USA). *P* values < 0.05 were considered statistically significant.

## Results

### Participants

**Fig. 1:**
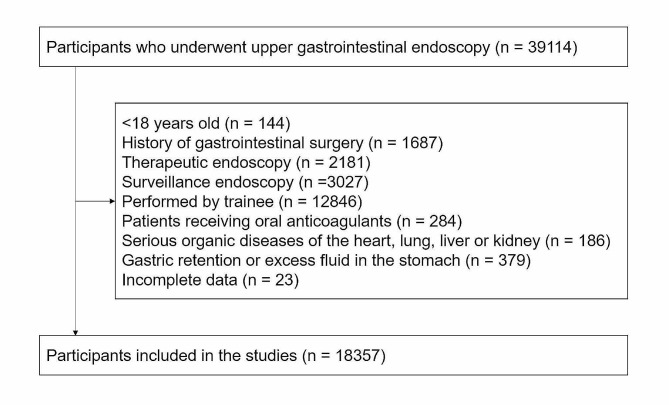
Flow chart of the study

A total of 39,114 participants underwent EGDs during the study period. Among them, we excluded EGDs from patients younger than 18 years (*n* = 144), patients with a history of gastrointestinal surgery (*n* = 1687), therapeutic endoscopy (*n* = 2181), surveillance endoscopy (*n* = 3027), patients receiving oral anticoagulants regularly (*n* = 284), patients with serious organic diseases of the heart, lung, liver or kidney (*n* = 186), gastric retention or excess fluid in the stomach (*n* = 379), performed by trainee (*n* = 12,846), incomplete data (*n* = 23) (Fig. 1). A total of 18,357 EGD examinations were included in the study. The median age of patients was 49.0 (35.0,58.0) years, and 8650 (47.1%) were male. A total of 13,946 (76.0%) EGD were performed under sedation.

### Examination time and focal lesion detection rate

12,759 examinations (69.5%) did not collect biopsy specimens. Focal lesions were detected in 8,908 participants (51.5%). Mean examination time of EGD without biopsy was 4.5 min (range, 2–29 min). The cut-off times used were 5 min. 17 endoscopists were classified into the fast (mean duration, 4.7 ± 3.6 min), and 16 into the slow (mean duration, 7.11 ± 4.6 min) groups. There were considerable differences among endoscopists in mean examination time and detection rate of focal lesions (Table 1). Characteristics of the endoscopist, patient and EGDs in the fast and slow endoscopist groups are shown in Table 2.

**Table 1:** Number endoscopic procedures, examination time, and detection rates of focal lesion of each endoscopist

	Endoscopist	Expert	Number of EGD	Number of EGD without biopsy (N, %)	Mean examination time of EGD (min) (Mean ± SD)	Number of EGDs detected with focal lesions (N, %)
Endoscopists with a short examination time (fast endoscopists)	2	NO	310	235 (75.8)	3.5 ± 3.1	129 (41.6)
	4	YES	960	763 (79.5)	3.3 ± 2.9	394 (41.0)
	5	NO	559	436 (78.0)	4.3 ± 3.9	215 (38.5)
	8	YES	1098	803 (73.1)	4.6 ± 3.6	527 (48.0)
	9	YES	2685	1857 (69.2)	5.6 ± 4.1	1266 (47.2)
	11	NO	201	162 (80.6)	4.1 ± 2.5	53 (26.4)
	16	YES	180	115 (63.9)	5.5 ± 2.9	128 (71.1)
	17	NO	472	327 (69.3)	5.1 ± 3.0	190 (40.3)
	18	YES	1008	750 (74.4)	4.4 ± 3.0	479 (47.5)
	19	YES	371	233 (62.8)	5.4 ± 4.3	165 (44.5)
	21	YES	220	110 (50.0)	5.7 ± 3.8	124 (56.4)
	22	NO	275	130 (47.3)	5.9 ± 3.7	210 (76.4)
	24	YES	596	502 (84.2)	3.8 ± 3.7	277 (46.5)
	25	NO	578	293 (50.7)	5.4 ± 3.8	339 (58.7)
	28	YES	1806	1317 (72.9)	3.9 ± 3.0	851 (47.1)
	31	NO	574	428 (74.6)	4.9 ± 3.3	282 (49.1)
	33	YES	708	559 (79.0)	5.6 ± 3.6	322 (45.5)
Endoscopists with a long examination time (slow endoscopists)	1	YES	405	284 (70.1)	7.9 ± 4.8	227 (56.0)
	3	YES	533	311 (58.3)	7.3 ± 5.0	310 (58.2)
	6	YES	96	64 (66.7)	6.0 ± 4.0	33 (34.4)
	7	NO	433	273 (63.0)	6.7 ± 4.9	189 (43.6)
	10	NO	609	443 (72.7)	7.7 ± 4.3	322 (52.9)
	12	YES	322	254 (78.9)	5.8 ± 4.2	145 (45.0)
	13	YES	137	51 (37.2)	9.0 ± 4.4	94 (68.6)
	14	YES	333	225 (67.6)	8.8 ± 6.7	200 (60.1)
	15	NO	241	127 (52.7)	6.9 ± 3.7	128 (53.1)
	20	NO	259	149 (57.5)	7.9 ± 4.2	167 (64.5)
	23	NO	420	250 (59.5)	7.7 ± 3.4	226 (53.8)
	26	YES	638	469 (73.5)	6.1 ± 4.4	254 (39.8)
	27	NO	395	308 (78.0)	5.7 ± 2.9	173 (43.8)
	29	YES	212	110 (51.9)	7.6 ± 4.7	112 (52.8)
	30	NO	572	309 (54.0)	6.3 ± 4.2	315 (55.1)
	32	NO	151	112 (74.2)	8.9 ± 4.7	62 (41.1)
Total	33	18	18,357	12,759 (69.5)	5.5 ± 4.1	8908 (48.5)

EGD, Esophagogastroduodenoscopy

**Table 2:** Characteristics of the endoscopist, patient and EGDs in the fast and slow endoscopist groups

	Examinations by endoscopists with a short examination time (fast endoscopists) (*N* = 12,601)	Examinations by endoscopists with a long examination time (slow endoscopists) (*N* = 5756)	*P* value
Endoscopist			
Male, n (%)	6509 (51.7)	1885 (32.7)	< 0.001
Expert, n (%)	9632 (76.4)	2676 (46.5)	< 0.001
Patient			
Male (N, %)	5838 (46.3)	2812 (48.9)	< 0.001
Age, M (IQR)	50.0 (36.0,59.0)	48.0 (34.0,58.0)	< 0.001
Number of images, M (IQR)	58 (44,76)	59 (45,77)	< 0.05
Sedation (N, %)	10,000 (79.4%)	3946 (68.6%)	< 0.001
Biopsy specimen taken (N, %)	3581 (28.4%)	2017 (35.0%)	< 0.001

### Detection rate of focal lesions

Compared with fast endoscopists, the overall focal lesion detection rate was higher among slow endoscopists (47.2% vs. 51.4%, *P* < 0.001). The most important difference between fast and slow endoscopists is the detection rate of gastric lesions. The detection rate of gastric lesions in slow endoscopists was significantly higher than fast endoscopists (29.7% vs. 35.9%, *P* < 0.001). Slow endoscopists have a higher detection rate of atrophic gastritis, gastric polyps, and gastric submucosal tumors than fast endoscopists. Fast endoscopists have a high detection rate of intestinal metaplasia. In esophageal and duodenal lesions, there was no statistically significant difference in the detection rate of focal lesions between fast and slow endoscopists (esophageal lesions: 17.0% vs. 15.9%, *P* > 0.05; duodenal lesions: 10.5% vs. 11.1%, *P* > 0.05) (Table 3).

**Table 3:** Lesions detected in the fast and slow endoscopist groups

	Examinations by endoscopists with a short examination time (fast endoscopists) (*N* = 12,601)	Examinations by endoscopists with a long examination time (slow endoscopists) (*N* = 5756)	*P* value
Focal lesion, n (%)	5951 (47.2)	2957 (51.4)	< 0.001
Esophageal lesions, n (%)	2145 (17.0)	913 (15.9)	> 0.05
Barrett’s esophagus, n (%)	380 (3.0)	114 (2.0)	< 0.001
Benign tumors of the esophagus, n (%)	174 (1.4)	94 (1.6)	> 0.05
Esophageal cancer, n (%)	45 (0.4)	22 (0.4)	> 0.05
Reflux esophagitis, n (%)	1189 (9.4)	485 (8.4)	< 0.05
Esophageal candidiasis, n (%)	33 (0.3)	24 (0.4)	> 0.05
Esophageal ectopic gastric mucosa, n (%)	344 (2.7)	149 (2.6)	> 0.05
Hiatal hernia, n (%)	270 (2.1)	116 (2.0)	> 0.05
Esophageal diverticulum, n (%)	6 (0.0)	12 (0.2)	< 0.05
Gastric lesions	3746 (29.7)	2065 (35.9)	< 0.001
Gastric ulcer, n (%)	550 (4.4)	266 (4.6)	> 0.05
Atrophic gastritis, n (%)	1536 (12.2)	951 (16.5)	< 0.001
Atrophic gastritis with intestinal metaplasia, n (%)	293 (2.3)	80 (1.4)	< 0.001
Gastric polyps, n (%)	1754 (13.9)	954 (16.6)	< 0.001
Submucosal tumors of the gastric, n (%)	219 (1.7)	137 (2.4)	< 0.01
Gastric cancer, n (%)	90 (0.7)	44 (0.8)	> 0.05
Extra gastric compression, n (%)	24 (0.2)	19 (0.3)	> 0.05
Gastric diverticulum, n (%)	6 (0.0)	4 (0.1)	> 0.05
Duodenal lesions, n (%)	1322 (10.5)	637 (11.1)	> 0.05
Duodenal polyps, n (%)	90 (0.7)	62 (1.1)	< 0.05
Duodenal ulcer, n (%)	1076 (8.5)	514 (8.9)	> 0.05
Duodenal diverticulum, n (%)	51 (0.4)	20 (0.3)	> 0.05
Submucosal tumors of the duodenal, n (%)	164 (1.3)	77 (1.3)	> 0.05

**Fig. 2:**
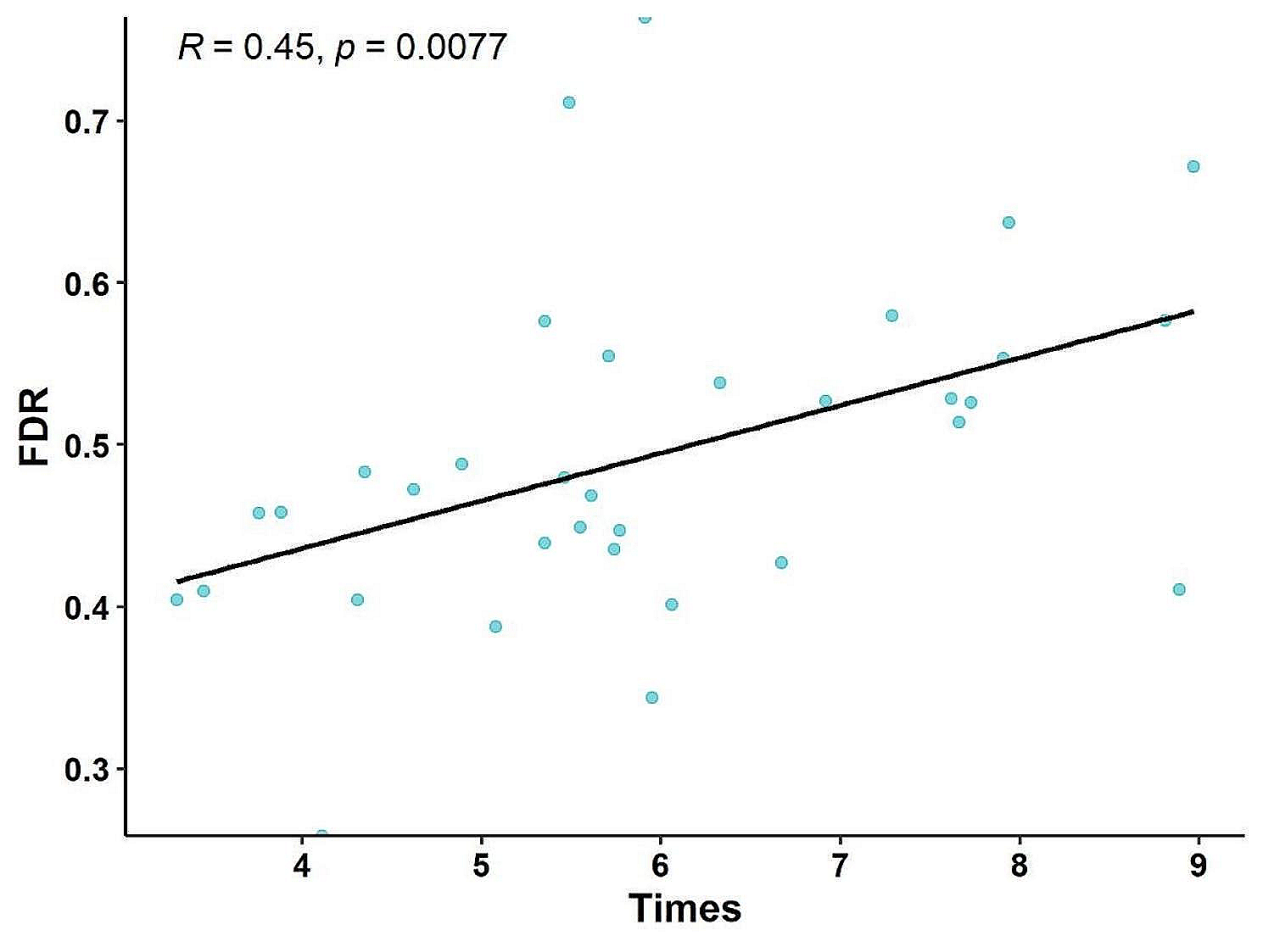
Relationship between mean observation time by endoscopists and detection rate of focal lesions (FDR) FDR, detection rate of focal lesions

In all EGD, Observation time is linearly related to focal lesion detection rate and biopsy rate (*r* = 0.252, *P* < 0.001; *r* = 0.428, *P* < 0.001). The focal lesion detection rate was strongly correlated with the biopsy rate (*r* = 0.354, *P* < 0.001). According to the average observation time and focal lesion detection rate of each endoscopist, it was found that the observation time was linearly related to the lesion detection rate (*r* = 0.449, *P* < 0.01) (Fig. 2).

### Factors associated with detection of focal lesions

In univariate analysis, observation time, patient age, male patient, non-expert, slow group endoscopist, biopsy rate, and number of images were significantly associated with focal lesion detection. Among them, the longer observation time and older patients are conducive to improving the detection rate of lesions. There was no statistically significant relationship between endoscopist sex, sedation and lesion detection rate. In multivariate analysis, observation time of more than 5 min, age > 40 years, male, non-expert, high biopsy rate, and more photographic recording were important factors in the detection of focal lesions (Table 4).

**Table 4:** Factors associated with detection of focal lesions

Variables		Focal lesions, n (%)	Univariate Analysis OR (95 % CI, *P* value)	Multivariable analysis OR (95 % CI, *P* value)
Times	Total		1.13 (1.12–1.14, *P* < 0.001)	1.04 (1.03–1.05, *P* < 0.001)
	< 5	3549 (39.8%)		
	[5,10)	3998 (44.9%)	2.26 (2.12–2.41, *P* < 0.001)	1.42 (1.31–1.53, *P* < 0.001)
	[10,15)	917 (10.3%)	3.00 (2.67–3.37, *P* < 0.001)	1.49 (1.30–1.70, *P* < 0.001)
	[15,20)	285 (3.2%)	4.23 (3.38–5.28, *P* < 0.001)	1.95 (1.52–2.50, *P* < 0.001)
	>=20	159 (1.8%)	3.27 (2.49–4.29, *P* < 0.001)	1.49 (1.10–2.02, *P* = 0.010)
Patient age	Total		1.04 (1.04–1.05, *P* < 0.001)	1.04 (1.03–1.04, *P* < 0.001)
	[18,40)	1920 (21.6%)		
	[40,60)	4244 (47.6%)	2.22 (2.07–2.38, *P* < 0.001)	1.84 (1.70–1.98, *P* < 0.001)
	[60,80)	2667 (29.9%)	4.47 (4.10–4.87, *P* < 0.001)	3.60 (3.28–3.95, *P* < 0.001)
	>=80	77 (0.9%)	9.11 (5.44–15.27, *P* < 0.001)	8.31 (4.86–14.18, *P* < 0.001)
Patient gender	Female	4270 (47.9%)		
	Male	4638 (52.1%)	1.47 (1.39–1.56, *P* < 0.001)	1.45 (1.36–1.55, *P* < 0.001)
Endoscopist gender	Female	4857 (54.5%)		
	Male	4051 (45.5%)	0.98 (0.93–1.04, *P* = 0.508)	
Expert	NO	3000 (33.7%)		
	YES	5908 (66.3%)	0.94 (0.88-1.00, *P* = 0.042)	0.92 (0.86–0.99, *P* = 0.028)
Group	FAST	5951 (66.8%)		
	SLOW	2957 (33.2%)	1.18 (1.11–1.26, *P* < 0.001)	0.98 (0.91–1.06, *P* = 0.590)
Biopsy	NO	4697 (52.7%)		
	YES	4211 (47.3%)	5.21 (4.86–5.59, *P* < 0.001)	3.66 (3.39–3.95, *P* < 0.001)
Sedation	NO	2122 (23.8%)		
	YES	6786 (76.2%)	1.02 (0.96–1.09, *P* = 0.522)	
Number of images	Total		1.01 (1.01–1.01, *P* < 0.001)	1.01 (1.00-1.01, *P* < 0.001)

OR, odds ratio; CI, confidence interval

**Fig. 3:**
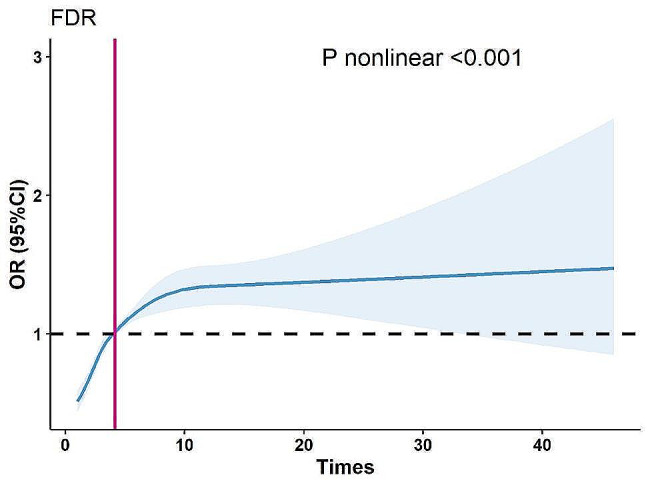
Relationship between observation time and detection rate of focal lesions (FDR). The odds ratio is represented by a solid line and the 95% confidence interval is represented by a shaded area. Adjusted restricted cubic spline models adjusted for patient age and gender, endoscopist gender, expert, biopsy, sedation, number of images FDR, detection rate of focal lesions

In restricted cubic spline regression, there was a significant nonlinear relationship between observation time and lesion detection rate (*p* for nonlinear < 0.001, Fig. 3). With the increase of observation time, the detection rate of lesions gradually increased until the curve stabilized after more than 10 min of observation time.

## Discussion

In this retrospective study of 18,357 endoscopic examination data, we found that the longer the observation time during endoscopy, the higher the detection rate of focal lesions. Compared with fast endoscopists (< 5 min), slow endoscopists (> 5 min) had a higher detection rate of focal lesions, especially in the detection of gastric lesions. In univariate and multivariate analyses, we found that observation time, patient age and gender, non-expert, biopsy rate, and number of images were important factors in lesion detection. There is a nonlinear relationship between observation time and lesion detection, and with the increase of observation time, the lesion detection rate continues to increase until the lesion detection rate tends to stabilize after more than 10 min. The results of this study are consistent with those of previously published articles [3, 10–12]. 

EGD is an important way to screen, diagnose and monitor gastrointestinal tumors. High-quality EGD can improve cancer detection rate and reduce cancer mortality. There is currently a lack of widely accepted and recognized endoscopic quality assessment protocols. Based on the currently published study, observation time is an important indicator of endoscopic quality [15, 16]. However, Current published studies have different definitions of observation time. In a retrospective analysis study of data from 111,962 participants, using a 3-minute cut-off time, more gastric adenomas or cancers were detected by slow endoscopists than by fast endoscopists (0.28% vs. 0.20%; *P* = 0.0054) [11]. The observation time for the study was defined as the time from the withdrawal of the endoscope from the second duodenal portion to the end of the EGD examination. This definition is similar to the definition of observation time in a colonoscopy. However, during endoscopic insertion, the endoscopist will observe the esophagogastric junction, antrum, pylorus, and duodenal bulb [17, 18]. Therefore, it is more accurate to use the time from insertion to endoscopic exit as the study metric compared with using endoscopic withdrawal time. Our study found that the slow endoscopists had a higher rate of lesion detection than the fast endoscopists, especially in the detection of gastric lesions. In the future, the fast endoscopists can extend the observation time of the stomach to obtain a higher lesion detection rate. In restricted cubic spline regression, it is found that there is a nonlinear relationship between observation time and lesion detection rate. Within less than 10 min, the detection rate of lesions increased with the increase of observation time, but after more than 10 min, the detection rate of lesions tended to stabilize. This suggests that longer observation time does not mean greater benefit. More research is needed to determine the cut-off time and whether there is an upper limit effect beyond which there is no benefit in lesion detection rates [19]. Slow endoscopists had a higher detection rate of atrophic gastritis while fast ones had a high rate of intestinal metaplasia. Endoscopic diagnosis of atrophic gastritis requires assessment of mucosal color and texture, appearance of submucosal blood vessels, and the architecture of the gastric rugae, followed by targeted examinations of focal abnormalities [20]. Therefore, slow endoscopists spend more time and have a higher detection rate. We found that fast group of experts has a great influence on the difference in the detection rate of atrophic gastritis with intestinal metaplasia. Expert can achieve high quality esophagogastroduodenoscopy with a high detection rate in short observation time (Suppl Table 1). However, it is difficult for non-experts to detect intestinal metaplasia quickly and accurately. Intestinal metaplasia requires pathological diagnosis and relies on biopsy material. Expert have extensive experience with biopsies and have a higher positive rate. Therefore, it is advisable to obtain a higher detection rate by increasing the observation time, especially for non-experts. In univariate and multivariate analyses, observation time, patient age, male, non-expert, biopsy rate, and number of images were related to lesion detection, while there was no statistically significant relationship between endoscopist’s sex, sedative use and lesion detection. We found that the top 4 endoscopists of number of EGD were all in the fast group, and all of them were expert. A large number of patients and high work pressure may result in decreasing in the detection rate of lesions. It differs from the conclusions of previous studies on Sedation. Sedation can significantly improve patient cooperation, satisfaction. The Asian consensus recommends the use of sedation to enhance the detection rate of superficial neoplasm of the esophagus and stomach [15]. A retrospective study suggested gastric polyps detection rate may be improved by inhibition of gastric muscle cramping with sedation [21]. Other studies have shown there was no statistically significant difference in the detection rate of precancerous lesions and early esophageal cancer between patients who underwent EGD screening with and without anesthesia assistance [22]. Sedation may improve the endoscopic detection rate of early cancer and high-grade intraepithelial neoplasia in the upper gastrointestinal tract probably through enhancing the use of accessary endoscopic techniques, prolonging observation time, and taking more biopsies in different locations [23]. 

There are several limitations in this study. First, this was a single-center, retrospective study. Second, a patient-selection bias might have been present. To reduce selection bias, we included all consecutive EGDs performed in a period where the staff of endoscopists was unchanged. Third, it is not possible to calculate the observation time of the esophagus, stomach, duodenum. The effect of biopsy time on observation time cannot be removed.

In conclusion, our study found that longer examination time improves the detection rate of focal lesions. Observation time is an important quality indicator of the EGD examination.

### Electronic supplementary material

Below is the link to the electronic supplementary material.

Supplementary Material 1

## Data Availability

The datasets analyzed during the current study are available from the corresponding author on reasonable request [Jun Fang, Email: xhfangjun@163.com].

## Abbreviations

EGD: Esophagogastroduodenoscopy
FDR: Lesion detection rate
