# Supplementary material for: Relationship between observation time and detection rate of focal lesions in Esophagogastroduodenoscopy: a single-center, retrospective study
Source: BMC Gastroenterol. 2024 Feb 15;24:75. doi: 10.1186/s12876-024-03157-3 (PMC10870502; doi:10.1186/s12876-024-03157-3)
Supplement: Supplementary file 1 — Supplementary Material 1 [file 12876_2024_3157_MOESM1_ESM.docx]

Suppl Table 1. detection rate of expert and no expert in the fast group.

|  | Expert | No expert | P value |
| --- | --- | --- | --- |
| Atrophic gastritis with intestinal metaplasia, n (%) | 253(2.6) | 40(1.3) | <0.001 |
